# Effect of Mat Moisture Content, Adhesive Amount and Press Time on the Performance of Particleboards Bonded with Fructose-Based Adhesives

**DOI:** 10.3390/ma15238701

**Published:** 2022-12-06

**Authors:** Catherine Rosenfeld, Pia Solt-Rindler, Wilfried Sailer-Kronlachner, Thomas Kuncinger, Johannes Konnerth, Andreas Geyer, Hendrikus W. G. van Herwijnen

**Affiliations:** 1 Wood K Plus-Competence Center of Wood Composites and Wood Chemistry, Kompetenzzentrum Holz GmbH, Altenberger Str. 69, A-4040 Linz, Austria; 2 Institute of Wood Technology and Renewable Materials, Department of Material Science and Process Engineering, University of Natural Resources and Life Sciences Vienna, Konrad-Lorenz Str. 24, A-3430 Tulln, Austria; 3 Fritz EGGER GmbH & Co.OG., A-3105 St. Pölten, Austria

**Keywords:** adhesives for wood, wood composite, internal bond strength, adhesion by chemical bonding

## Abstract

The study evaluates the performance of laboratory, single-layered particleboards made out of fructose-hydroxymethylfurfural-bishexamethylenetriamine (SusB) adhesive as a sustainable alternative. Several production parameters such as mat moisture content (MMC), adhesive amount and press time were varied and their effect on the bonding efficiency investigated. The internal bond strength (IB) and thickness swelling after 24 h of water immersion (TS) were taken as evaluation criteria for the bonding efficiency. pMDI-bonded particleboards were produced as fossil-based, formaldehyde-free reference. Particleboard testing was complemented by tensile shear strength measurements and thermal analysis. It was found that the MMC has the highest impact on the internal bond strength of SusB-bonded particleboards. In the presence of water, the reaction enthalpy of the main curing reaction (occurring at 117.7 °C) drops from 371.9 J/mol to 270.5 J/mol, leading to side reactions. By reducing the MMC from 8.7%, the IB increases to 0.61 N/mm^2^, thus surpassing P2 requirements of the European standard EN312. At a press factor of 10 s/mm, SusB-bonded particleboards have a similar IB strength as pMDI-bonded ones, with 0.59 ± 0.12 N/mm^2^ compared to 0.59 ± 0.09 N/mm^2^. Further research on the improvement of the dimensional stabilization of SusB-bonded PBs is needed, as the TS ranges from 30–40%.

## 1. Introduction

Particleboards are an important value-added panel product in the wood-based industry with a wide range of application, for example, in furniture (66%) or construction (27%) [1,2]. Currently, formaldehyde-based adhesives still hold a dominant position in the wood panel industry in the manufacture of particleboards [3,4], among others. The utilized formaldehyde and especially the emissions thereof have become a subject of concern over the years. This sparked research of low-emitting adhesive compositions [5,6]. The reclassification of formaldehyde in 2014 partly shifted the focus in the adhesive research and put it on the development of formaldehyde-free adhesives [7,8]. The main advantage that boosted the surge of those formaldehyde-based binders resides in their high applicability in relation to their excellent bonding performance and the quality and strength of the boards. For this reason, the production parameters, such as pressure profile and temperature or curing time are optimized in the panel manufacturing industry for the performance of formaldehyde-based adhesives, which is often a clear disadvantage for new adhesive developments [3]. A synthetic, fossil-based adhesive alternative to amino resins like urea formaldehyde that is already in industrial use in Oriented Strand Board (OSB) production and to a lesser extent in particleboard (PB) production is polymeric methylene diphenyl diisocyanate (pMDI) [9]. pMDI is the most obvious formaldehyde-emission-free candidate due to the low resin dosage, extreme moisture resistance, low swelling and high strength of pMDI-bonded boards [10]. Even though pMDI has already been in use since 1973, its market share as the sole adhesive system in particleboard in Europe is approximately only 1% of the total production volume [11]. The necessity of hardware modifications in the production process, e.g., adaptation to a lower adhesive spread rate, safety measures or the required use of a releasing agent, as well as the higher adhesive cost and uncertainties in the pMDI supply chain, have restricted its utilization in particleboard production so far [9].

A recent technological evaluation of the performance of formaldehyde-free adhesives for lab-scale particleboards concluded that except for pMDI-based systems, most of the alternatives have a considerably reduced curing reactivity [9]. However, at the industrial scale, the curing of pMDI is also slower than that of conventional aminoplast adhesives. In particleboard manufacture, a highly reactive adhesive is needed for a sufficient cure in the core layer of the board, where the temperature is limited to below 120 °C [12]. The problem with a reduced reactivity is twofold. The rate at which the bonding strength develops during curing critically affects the production costs as well as the performance of the particleboards [9]. If the adhesive in the core layer has a lower cure speed due to temperature restrictions, longer hot-pressing times are needed to reach the standard requirements (e.g., internal bond strength), resulting in an overall reduced production throughput and consequently higher production costs. The aforementioned internal bond strength (IB) is an important quality parameter of particleboards in this context. It is measured as the tensile strength perpendicular to the plane of a particleboard, and considering the typically lower density in the core layer, the IB usually indicates the strength of the core layer and as such provides insights into the adhesive performance. An indicator of the efficiency of the production process is the press factor. It is defined as the time needed to cure 1 mm of panel thickness and as such is related to the production process itself. Standard UF-bonded particleboards only need press factors as low as 3–7 s/mm on an industrial scale and somewhat higher press factors of 5–12 s/mm in laboratory production using 10% adhesive (based on dry wood) and typical hot-pressing temperatures of 180–240 °C [9]. Typical press factors for pMDI-bonded particleboards at laboratory scale lie slightly higher at 10–15 s/mm, but lower adhesive amounts of only 2–6% (based on dry wood) are needed [13].

However, in light of regulations limiting formaldehyde and volatile organic compound (VOC) emission associated with different stages of the life cycle of wood panels [4,8], it has become a necessity to develop suitable formaldehyde-free alternative adhesives [7,14,15]. Research on renewable, bio-based adhesives is an extremely active field aiming to provide a solution from a bioeconomy perspective [16,17]. For example, numerous promising wood adhesives were recently reported based on natural biopolymers such as lignin [18,19], tannin [20,21], proteins [22,23], starch [24,25], cellulose derivatives [26] or other carbohydrates [27]. However, comparable to the success story of formaldehyde-based adhesives [28], the potential adoption of bio-based adhesives in the wood-based panel market will be driven by their functional performance and not merely by the green credentials of renewable adhesives [29].

In an earlier work [30], a carbohydrate-based adhesive formulation for a cleaner production of interior particleboards (EN312, P2 classification [31]) was reported. It was found that bio-based Hydroxymethylfurfural (HMF) acted as a key reactant and increased the reactivity and performance of fructose-bishexamethylenetriamine (BHMT) adhesives. Three prototype particleboards were produced under non-optimized conditions as a proof of concept. The obtainable press factor, 20 s/mm, was among the best of previously reported HMF-based adhesives, but not yet competitive with UF-bonded or pMDI-bonded particleboards, as the production parameters were not optimized.

There is a large body of literature on the relation between processing conditions and the physical properties of particleboards bonded with conventional urea-formaldehyde adhesives [12]. It is widely reported that the type of UF resin (its efficiency and amount) and the processing parameters (such as mat moisture content, press time, pressure and temperature) influence the physical properties and mechanical strength [12]. 

In this study, we wanted to test the hypothesis that the optimization of processing conditions such as mat moisture content, adhesive amount and press factor would result in an improved IB strength of SusB-bonded particleboards, making them competitive with UF and pMDI systems. Certain production parameters, such as a press temperature of 220 °C and a maximum press factor of 12 s/mm, are predetermined by the standard operating conditions used in the manufacture of UF (E1-type)-bonded particleboards and result from techno-economic considerations [9]. Based on earlier work, the most efficient SusB adhesive composition in terms of cure speed is fructose/HMF/BHMT with a molar ratio of 3.7/0.2/1. An objective of this work is to further evaluate the efficiency of this adhesive composition in terms of adhesive amount and press time. The performance of particleboards was determined by IB testing and thickness swelling after 24 h of water immersion and compared to a pMDI reference and literature values of UF. Overall, the aim of this work is to evaluate the potential of SusB-adhesive as a sustainable alternative in P2 particleboards and identify potential adjustments for future research avenues. 

## 2. Materials and Methods

### 2.1. Raw Materials

Industrial wood particles for the core layer were used for the manufacture of the particleboards. The industrial wood particles (particle size 0.2–8 mm) were obtained from an Austrian particleboard manufacturer (FRITZ EGGER GmbH & CO. OG, Unterradlberg, Austria). The industrial wood particles had a recycling content of 35% and mainly consisted of spruce (*Picea abies*) and pine (*Pinus*) and to a small extent poplar (*Populus*). The particle size distribution is given in the Supporting Information. The particleboards were produced in two press series. The moisture content of the wood particles was constant within each press series (1.8% or 3%, respectively, see Supporting Information). Fructose solution FF95 (70.5% dry solids, 95% purity) was supplied by Cargill Deutschland GmbH (Krefeld, Germany). An aqueous HMF solution (comprising 5.5 wt.% HMF, 54.5 wt.% residual sugars) was produced from a diluted fructose solution FF95 (60% *w/w*) in a pressurized batch reactor [32]. Bishexamethylenetriamine (BHMT, crystalline, high purity) was purchased from Sigma Aldrich (St. Louis, MO, USA). 

### 2.2. Adhesive Synthesis

The procedure for the synthesis of the fructose/HMF/BHMT (SusB) adhesive was already described in detail in Rosenfeld et al. [30]. Fructose solution FF95 and HMF precursor were added to a three-neck flask, which was equipped with a thermos element and a reflux condenser. The HMF precursor contained 0.1 wt.% of sodium dithionite, which was added during the production of the HMF as a stabilizer. The FF95 and HMF mixture was heated to 40 °C with a hot plate magnetic stirrer with Teflon coating. When the reaction temperature reached 40 °C, the first half of molten BHT was added, and the reaction mixture was further heated to 60 °C. The reaction was kept for 60 min at 60 °C. The adhesive was synthesized one day prior to the production of the particleboards and stored in the refrigerator at 7 °C. Directly before adhesive application, a second portion of BHMT (10 wt.%) was added to the formulation in order to obtain a molar ratio of fructose/HMF/BHMT = 3.7/0.2/1 in the final resin. The solid content of the glue was 55%, as determined by oven drying of 2 g of adhesive at 120 °C for 2 h in an aluminum tray. The viscosity of the adhesive after synthesis is typically about 390–400 mPas measured at 23 °C using a cone plate system with a diameter of 50 mm and a cone angle of 1° on an MCR 302 rheometer (Anton Paar GmbH, Graz, Austria). 

### 2.3. Tensile Shear Strength (ABES)

The reactivity of the SusB adhesive was initially investigated by measuring the development of tensile shear strength after hot-pressing. More specifically, it was used to investigate the influence of press temperature on the cure speed and strength development. The tensile shear strength was determined with an automated bonding evaluation system (ABES) equipped with an air-cooling device [33]. Testing was done according to ASTM-D7998-15 [34]. For these preliminary tests, uniform, planar and defect-free European beech (*Fagus sylvatica*) veneers with dimensions of 120 × 20 × 0.59 mm were used. These veneers were acclimatized prior to testing at 20 °C with 65% relative humidity. 125 g/m^2^ adhesive were used in the testing, and press temperatures of 105 °C or 120 °C were applied, respectively. A 60 s cooling phase was used before applying a tensile force to reduce any thermoplastic influence on the testing results. The hot-pressing time was varied in order to obtain the cohesive strength development curve of the adhesives. 

### 2.4. Thermal Analysis

Differential scanning calorimetry (DSC) was used for investigating and characterizing the thermal behavior of the adhesives. More specifically, it was used to study the effect of different water content on the curing of the SusB adhesive. Measurements were performed with a Polyma 214 (Netzsch-Gerätebau GmbH, Selb, Germany); 2–5 mg of uncured adhesive samples was measured in a closed, high-pressure steel crucible. The device was calibrated by measuring samples of gallium, indium, tin and bismut at a heating rate of 5 K/min. Typical measurements were performed from 20 °C to 350 °C. The data were analyzed using Netzsch Proteus^®^ software Version 8.0 (Netzsch-Gerätebau GmbH, Selb, Germany) and Origin 2016G (OriginLab Corporation, Northampton, MA, USA). 

### 2.5. Particleboard Production

Particleboards with dimensions of 450 × 500 × 16 mm were pressed in a laboratory press (G. Siempelkamp GmbH &CO. KG., Krefeld, Germany) using a pressure of 3.5 N/mm^2^ and a hot-pressing temperature of 220 °C. The adhesive was sprayed onto the particles with an industrial spraying system in a ploughshare mixer to ensure good adhesive distribution, and the resinated particles were manually distributed into a pressing mold. After hot-pressing at 220 °C, the test boards were conditioned for 10 days at a standard climate of 20 °C and 65% relative humidity. The target density of the particleboards was 650 kg/m^3^.

With the aim of investigating the effect of press factor and mat moisture content on the particleboard performance, two series of experiments were done as presented in Table 1 and Table 2. Two particleboards were made for each parameter variation. pMDI was selected as a formaldehyde-free reference. In general, the initial mat moisture content in percent is calculated from the mass of water in the board divided by the total mass of the formed particleboard mat (dry basis). The amount of water in the board is the sum of water in the adhesive, moisture of the wood particles and, if applicable, any additionally added water (see Table 1).

The total mass of the dry wood is considered as 100%, and based on this the desired amount of adhesive (based on solid content) is added.

In the production of SusB-bonded particleboards with higher mat moisture content (8.7–12.0%, see Table 1), additional water was added to the SusB adhesive, and the diluted binder was sprayed onto wood particles. For the pMDI-bonded boards, the adhesive was sprayed first, and water was added in a separate step afterwards. The added water is given in weight percent based on the dry weight of the particles. No additional water was added in press series 2; the decreased mat moisture content was achieved by utilizing a lower adhesive amount (from 4.5% to 10.0%, see Table 2). 

In total, there are two boards in each press series, which were pressed with the same settings (adhesive amount=10.0%, press factor = 14 s/mm). The mat moisture content in these boards differs slightly, with 8.7% (Press series 1, Table 1) and 7.7% (Press series 2, Table 2) due to differences in the moisture of the wood particles.

### 2.6. Particleboard Testing

After board manufacturing, the samples were cut to individual sample size and conditioned to constant mass in an atmosphere with a relative humidity of 65% and a temperature of 20 °C.

#### 2.6.1. Board Density

The density of the particleboards was evaluated by mathematical means as the ratio of the mass of test specimen to its volume. Ten test specimens were prepared for each particleboard and used to estimate the density of the whole board. The density determination was done according to EN323 [23] at a sample size of 50 × 50 mm. A potential vertical density gradient of the produced particleboards is not considered, but the Pearson correlation coefficient between internal bond strength (IB) and density was calculated for all particleboards (Supporting Information).

#### 2.6.2. Internal bond Strength

The determination of internal bond strength perpendicular to the plane (IB) was conducted according to EN319 [35] on a universal testing machine (Shimadzu Europe GmbH, Duisburg, Germany). Each test piece was then bonded to metal loading blocks using a hot-melt glue. The testing assembly was further placed in the machine, and force was applied until rupture occurred.

#### 2.6.3. Thickness Swelling

The thickness swelling of all particleboards was determined after 24 h of immersion in water according to EN317 [36]. Prior to testing, the produced test specimens were stored at a standardized climate (20 °C, 65% relative humidity). The thickness and weight of each test piece (50 × 50 mm) was measured at the intersection of the diagonals before and after immersion in water, which relates to the thickness swelling.

#### 2.6.4. Statistical Evaluation

Statistical analysis of the results was carried out in Origin 2016G (OriginLab Corporation, Northampton, MA, USA). 

The Pearson correlation coefficient is a nonparametric measure and was used to test the linear association for two variables. Pearson coefficients close to zero indicate a low association, whereas a Pearson coefficient close to −1 or +1 is associated with a strong linear correlation of two parameters.

In addition, Tukey’s post hoc test was used to analyze pairs of means and test if there was a significant difference. The confidence level was 95%.

## 3. Results and Discussion

### 3.1. Statistical Evaluation

**Figure 1:**
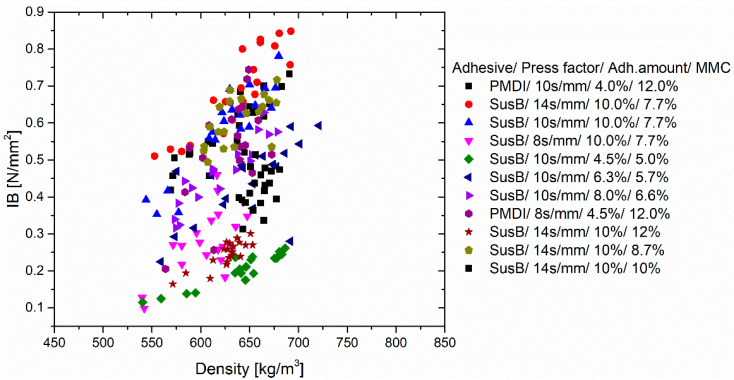
Correlation between IB and density determined for each set of particleboards.

It is well-known that the internal bond strength correlates with the density of the board [12]. Thus, it must be ensured that the potential IB differences do not result simply from density variations. The Pearson coefficient was calculated to evaluate the correlation between the IB and density of each set of particleboards that was made under the same pressing conditions (Adhesive type/Press factor/Adhesive amount/Mat moisture content). In general, a linear correlation can be seen in Figure 1. The determined Pearson coefficients are given in the Supporting Information (Table S1). 

The overall trend in Figure 1 is expected, as higher densities imply a better contact between the adhesive-coated particles [37]. Therefore, it is necessary that the densities of the individual particleboards do not significantly differ from each other. This can be evaluated by using a post hoc test, such as Tukey’s test, that compares the means of the particleboards’ densities (Supporting Information, Figure S1) and finds significant differences at a confidence level of 95% (Supporting Information, Table S2). At this confidence level, it was found that only three board variations significantly differ from each other in the following parameters (Adhesive/Press factor/Adhesive amount/MMC):

- SusB/14 s/mm/10%/10% and SusB/10 s/mm/8.0%/6.6%
- SusB/14 s/mm/10%/10% and SusB/8 s/mm/10.0%/7.7%
- SusB/8 s/mm/10%/7.7% and SusB/10 s/mm/6.3%/5.7%

The differences in densities and the resulting influences on particleboard performance must be taken into account when the aforementioned variations are compared. A comparison of these combinations is, however, of minor interest regarding the research questions investigated in this work. 

### 3.2. Adhesive Performance

#### 3.2.1. Cure Temperature

**Figure 2:**
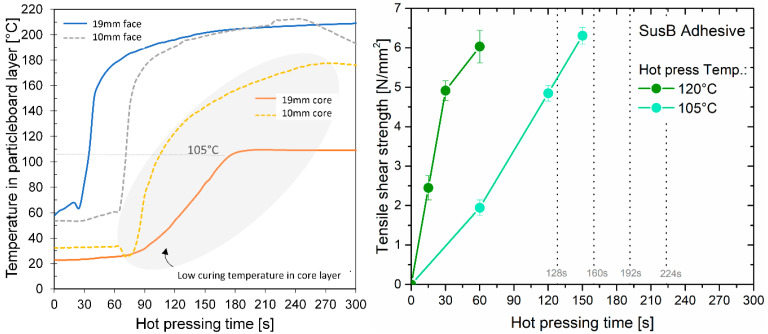
Left: Temperature profile in core and face layer of UF-made PBs during hot-pressing at 220 °C [9], Right: Tensile shear strength development of fructose-HMF-BHMT adhesive (SusB) pressed at 105°C and 120°C (adapted from [30]).

As previously reported in the literature [38], the formation of water vapor and the resulting gas pressure restrict the temperature of the particleboard’s core layer to below 120 °C. Figure 2 (left plot) shows that the core temperature of a 19 mm particleboard remains constant at about 100 °C for a certain time when hot-pressed at 220 °C. This temperature plateau was already reported by Maku et al. [38], who studied the temperature and moisture distribution in particleboards during hot-pressing. They analyzed the temperature profile for PBs with an MMC ranging from 11% to 30% and found that the higher the initial MMC, the longer the time span of this constant temperature plateau at 100 °C [38]. This restriction of low temperatures in the core layer makes a high curing efficiency of the adhesive in the highlighted range (Figure 2, left plot) extremely important. A high press temperature of 220 °C and press pressure of 3.5 N/mm^2^ was used in the particleboard production to enable a fast temperature development in the core layer.

In the strength development of the SusB adhesive, ABES was the primary system for conducting application and composition testing. An initial assessment of the adhesive performance was done by measuring the tensile shear strength after hot-pressing at 105 °C and 120 °C as a function of press time (Figure 2, right plot). The hot-pressing temperature of 105 °C was chosen based on the temperature profile in the core layer of a particleboard (Figure 2, left plot). Tensile shear strength measurements at 120 °C were conducted to highlight the impact of slightly increased temperatures on the curing reaction. The development of the resulting bond strength gives information about the adhesives cure speed, and based on that, a rough estimation can be made whether or not the adhesive will be sufficiently cured within the defined press time in the particleboard manufacture. In general, the cure time is predetermined by the press factor. The corresponding time limit for a press factor of 8, 10, 12 and 14 s/mm is given in Figure 2 (right plot) for a 16 mm particleboard, resulting in press times of 128 s, 160 s, 192 s and 224 s. The maximum obtainable tensile shear strength is limited by the strength of the beech veneers, as values above 6 N/mm^2^ are dominated by wood failure [39]. Figure 2 (right plot) shows that the SusB adhesive has a high cure speed. The findings indicate that the sample is sufficiently cured at a temperature of 105 °C within 150 s. However, several other factors influence particleboard performance, such as moisture content, density of the core layer of the boards, particle size and distribution as well as particle geometry and properties [9,40]. Even though the utilized wood veneers were stored in a standardized climate prior to testing, there is a significant reduction in the moisture content of these thin veneers when they come into contact with the hot-pressing plates of the ABES test machine. Thus, the measured tensile shear strength values simulate a very dry curing process, which clearly differs from actual particleboard manufacturing processes. Therefore, the obtained results must also be verified with actual laboratory press runs and subsequent IB testing of particleboard samples. This is the only way to identify side effects during curing, e.g., curing delay due to impeded water vapor diffusion. 

#### 3.2.2. Cure Reactions

In general, a complex polymeric structure is formed upon curing of the SusB adhesive. A more thorough discussion of potential structural elements can be found in the previous publication [30]. However, it must be emphasized that a potential curing mechanism is not proposed. The complex mechanism of Maillard-type reactions is still a controversial and ongoing research topic [41]. Possible occurring reactions in the SusB curing are, for example, the self-condensation of HMF, reactions of HMF and the amine crosslinker BHT as well as the formation of fructosylamines and further Maillard-type products. The relation between the SusB material response and its chemical structure is still a missing link. A detailed chemical analysis is an important task for future work, as a proposal for a reaction mechanism can only be made after a more rigorous analysis, for example with ^13^C- or ^15^N-selective labelling. 

**Figure 3:**
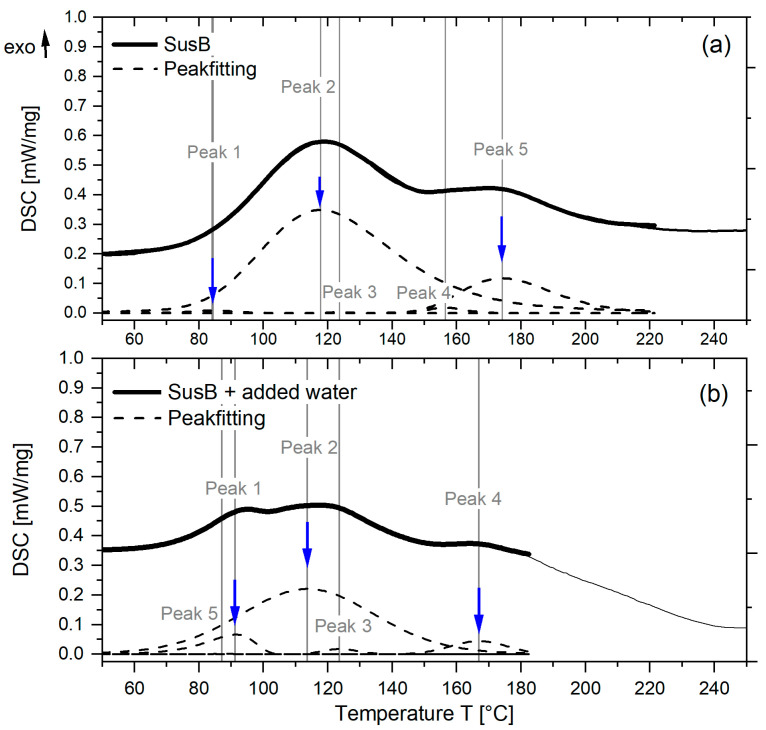
Thermal analysis of SusB adhesive without added water (**a**) and with 43.85 wt.% water added based on dry weight of adhesive (**b**).

Still, DSC measurements were carried out in order to study the curing reaction of the SusB adhesive and determine the effect of higher water contents on the reaction progression. The deconvoluted DSC peaks in Figure 3 (upper plot) already show that several reactions are occurring, although it is not clear what types of reaction are taking place. For the adhesive studied in Figure 3 (lower plot), the same amount of water was added as in the manufacture of the particleboards with an MMC of 12% (neglecting wood moisture content). However, it must be considered that water cannot evaporate during DSC measurements in closed crucibles. The reaction enthalpy in Figure 3 is calculated based on the dry weight of the adhesive in order to take the differences in solid content into account. In a previous study [30], we showed that the second peak (Peak T = 117.7 °C, Figure 3 upper plot) is influenced by the amount of amine crosslinker available for the curing reaction. Considering the temperature range, this peak also has the highest contribution to the curing reaction in the core layer of a particleboard. 

The lower plot of Figure 3 shows the changes in the curing reactions when additional water is added to the SusB adhesive. A clear decrease in the reaction enthalpy can be seen for the aforementioned Peak 2 from 371.9 J/g to 270.5 J/g, whereas an increase is noted for Peak 1 (see Table 3). In general, reaction peaks with a very low enthalpy (<6 J/g) are not depicted in Figure 3, but for the sake of completeness, they are included in Table 3.

This is a surprising finding, as it suggests that in the presence of water the thermodynamic equilibrium between the individual curing reactions is shifted, which might affect the overall adhesive performance as well. To our knowledge, this observation has not been reported for fructose-based adhesives before. As the reaction mechanism of the curing has not been studied yet, any suggestions for the occurring side reactions in the presence of water would be highly speculative.

It was, however, already verified that the curing kinetics of pMDI are significantly affected by moisture. In general, the reaction enthalpy and reaction rate of pMDI increases with increasing moisture content up to about 12% [42]. At higher MMC, the reaction kinetics remain almost unchanged; built on these findings, an MMC of 12.0% was chosen for pMDI reference boards. This contrasting response of pMDI and SusB adhesive to moisture is not surprising, as the chemistry of the underlying curing reactions differs as well.

### 3.3. Particleboard Test Results

#### 3.3.1. Variation of Mat Moisture Content

There have been numerous studies on the heat transfer into the fiber or particle mat, its relation to target density and moisture content as well as its effect on UF curing during hot-pressing [12]. In an early publication, Maku et al. [38] studied the temperature and moisture distribution in particleboards during hot-pressing. Among other findings, they drew two important conclusions: They found that when the initial conditions (hot plate temperature, moisture distribution) are symmetrical about the center layer, the resulting cure temperature distribution is also symmetrical about the center. In addition, they concluded that in terms of evaluating the curing adhesive, the most important layer is the core layer, as its temperature rises the most slowly during hot-pressing. Building on these conclusions, only single-layered particleboards were produced within this study for evaluating the adhesives’ efficiency under different processing conditions. We want to investigate in this study whether the initial mat moisture content has a direct or indirect influence on the SusB curing that reflects on the particleboard’s performance. Studies on the heat transfer during hot-pressing were not replicated in this work. Even though the thermal conductivity coefficient of the SusB adhesive might differ from UF, it is reasonable to assume that the heat transfer mechanism works in the same way.

**Figure 4:**
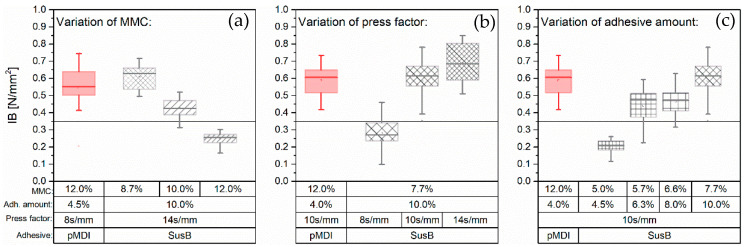
Internal bond strength of single layered particleboards with the minimum value for P2 classification (P2 = 0.35N/mm^2^, EN 312 [31]) indicated as horizontal line. (**a**) Variation of mat moisture content (MMC), (**b**) Variation of press factor, (**c**) Variation of adhesive amount, *n* = 20.

Several works showed that the bonding efficiency of UF is strongly influenced by the MMC [12]. It is well-known that the types of reactive species participating in the UF curing reaction depend on the reaction conditions such as temperature, pH and reaction time [43]. In an earlier study, Maku et al. [38] investigated the influence of the MMC on the temperature profile in the core layer of a UF-bonded particleboard. They could show that core temperatures above 100 °C are reached earlier for particleboards with lower MMC. This is essential, as it directly affects the UF curing reactions. At low temperatures and weak acid pH, the formation of methylene-ether bridges (-CH_2_-O-CH_2_-) is favored, whereas at higher temperatures and lower pH more stable methylene bonds (-CH_2_-) are formed. It is thus not surprising that there is an optimal MMC range for UF-bonded particleboards. For maximizing IB in boards with uniform MMC, it seems to be about 10% for particleboards [12,40], 12–15% for low-density fiberboards and 13–14% for medium-density fiberboards [44]. Again, a higher MMC leads to a reduction in the IB strength of UF-bonded particleboards. In pMDI-bonded boards, the optimum MMC is about 12% [42]; at higher values no significant change in the performance is observed. In the first press series with SusB adhesive, the mat moisture content (MMC) was varied, and three sets of particleboards with an MMC of 8.7%, 10.0% and 12.0% were prepared. For the particleboards with an MMC of 10.0% and 12.0%, additional water was added to the adhesive prior to particleboard production. Figure 4 suggests that the added water has a negative impact on the particleboard performance, reducing the mean IB from 0.61 ± 0.06 N/mm^2^ (MMC = 8.7%) to 0.25 ± 0.04 N/mm^2^ (MMC = 12.0%).

From a manufacturing perspective, it is interesting to note that the resinated chips have a pronounced cold tack when the SusB adhesive is used. This effect is slightly time-dependent, as the cold tack decreases the longer the resinated chips are resting in a ventilated area. This might result from drying processes. On the other hand, it was observed that cold tack is reduced when significantly higher amounts of water are present (MMC=12.0%). Unfortunately, a higher MMC affects the mechanical properties of the boards negatively (Figure 3, left plot). Still, future investigations on an optimum cold tack are an important task with regard to potential industrial application of the adhesive. A sufficient cold tack is needed during production to ensure that the resinated particles stick together and can be transported without particle-size-derived wood chip fractionation, but too high a cold tack could block the chip-spreading machines in the industrial plants. In general, the cold tack was lowest for the boards with 4.5% adhesive amount. The less resin used, the lower the cold tack. Resinated particles were almost dry for 4.5% resin, with cold tack that is comparable to the pMDI variant. 

In general, prior studies on UF [12,38] already indicate that the initial MMC can directly or indirectly influence the particleboard performance, e.g., by surface densification, by shifting the equilibrium of the (condensation-based) curing reaction or by reducing the core temperature of the board. The results from tensile shear strength testing (Figure 2, right plot) show that the SusB adhesive cures more rapidly at higher hot-pressing temperatures. Consequently, a lower MMC is beneficial for the curing of the SusB adhesive in the utilized laboratory press, as higher core temperatures are reached faster. It is difficult to transport these findings to industrial scale, as the water steam cannot escape in the industrial hot-presses, which affects the heat transfer and the obtained temperatures within the board.

However, it is important to recognize that the mat moisture might also influence the adhesive’s cure speed directly. When water is removed, e.g., by evaporation, the equilibrium position of this condensation reaction is driven to the condensation product, and the curing of the adhesive is thermodynamically favorable. For a higher initial MMC, it takes longer to evaporate the excess water from the system and thus could slow down the reaction rate. The main limitation in this regard is that the reaction mechanism of the curing SusB adhesive is still unknown. Nevertheless, it is possible to gain insights into the curing reactions from thermal analysis and the evaluation of the reaction enthalpy. DSC showed that the competing, low-temperature reaction at 91.3 °C (Peak 1, Figure 3 lower plot) is favored when more water is present during the curing. This can be seen by an increase in the reaction enthalpy from 2.7 J/g to 33.7 J/g. In contrast, the reaction enthalpy of the main curing reaction at 113.7 °C decreased from 371.9 J/g to 270.5 J/g. These findings suggest that for fructose/HMF/BHMT adhesive, an increased water content also directly influences the curing reactions. Considering the reduced IB values in PBs with higher mat moisture content, it can be concluded that the increase in the low-temperature curing reaction (Peak 1) is not beneficial for the strength development of the adhesive. Within the studied MMC range, the best results were obtained with an MMC of around 8.7% for SusB-bonded particleboards, which is lower compared to pMDI-bonded ones (12%) [42] or UF-made particleboards (10%) [40]. In general, the mat moisture content can only be varied within a limited range when the adhesive amount and solid content are kept constant.

Overall, in commercial production, it is more common to produce three-layered particleboards. Often in these commercial systems the initial moisture content of the face and core layers differs. Typically, the moisture content of the face layer is higher than in the core layer. The reason for this is that upon heating, the diffusion of water vapor is comparatively easy, and it can be considered that the high-temperature vapor of the face layer diffuses into the core layer of lower temperature because of the impossibility of surface evaporation. Thereby, the heat conduction is accelerated, and the apparent thermal diffusivity is considerably increased, leading to remarkably shortened press cycles. Future research should consider the potential effects of moisture content on the curing of SusB adhesive in three-layered particleboards more carefully. For example, it can be expected that even if the three-layered particleboards are prepared to the same mean moisture content, their temperature behavior is greatly influenced by the initial moisture distribution in the face and core layers. Of course, the temperature distribution in these layers is also influenced by the thickness ratio of the three-layer construction. Further in-depth research on SusB-bonded three-layered particleboards is needed to transfer the findings from this study to a more commercial application in three-layered particleboards. 

#### 3.3.2. Variation of Press Time

A relatively high press factor (14 s/mm) was used for studying the effect of mat moisture content. A further reduction in press time is desired in order to further minimize the overall production costs [9]. The tensile shear strength development of the SusB adhesive was determined after hot-pressing at 105 °C (Figure 2, right plot). The temperature was chosen based on the temperature profile in the core layer of a particleboard. The results indicate that theoretically a sufficient strength should be reached for PBs produced with 10 s/mm. Figure 4 (middle plot) shows that the preliminary ABES results are in good accordance with the obtained internal bond strength of particleboards produced at 14 s/mm, 10 s/mm and 8 s/mm. Only the PB made at 8 s/mm did not reach the benchmark value of 0.35 N/mm^2^, which is necessary for a P2 classification according to EN312 [31]. This is somewhat expected, as the ABES results suggest that the strength development is not sufficient, and the maximum tensile shear strength is also not reached within this low press time (Figure 2**,** right plot). Thus, optimization of the IB at a press factor of 8 s/mm must start by further enhancing the adhesives’ cure speed rather than production variables. 

The cure speed of the pMDI reference boards showed no significant change in the IB for 10 s/mm or 8 s/mm, with 0.59± 0.08 N/mm^2^ and 0.54 ± 0.13 N/mm^2^, respectively. On this note, it should be considered that an additional 0.5% of adhesive was used for the 8 s/mm boards. This corresponds to a 12.5% increase in adhesive amount compared to the 10 s/mm boards. Nevertheless, the good performance of the pMDI reference must be positively emphasized.

#### 3.3.3. Variation of Adhesive Amount

The previous findings highlight that the initial MMC significantly affects the resulting internal bond strength of particleboards (Figure 3, left plot). Without changing the solid content of the SusB adhesive (57%), the lowest resulting MMC is 7.7–8.7%, depending on wood moisture. Nevertheless, a further reduction in MMC can be obtained by lowering the adhesive amount utilized in the PB production. With regard to UF and pMDI adhesives, Papadoploulus [40] studied the influence of adhesive amount and selected process variables (mat moisture content, wax content, press temperature) on mechanical properties. His findings clearly showed that as the adhesive content increases, all strength properties of the board increase as well. This relation can also be seen for the SusB adhesive, as the IB increased with adhesive amount (Figure 3, right plot). From an economic and ecological perspective, the adhesive amount should be optimized in a way that the lowest possible adhesive amount is used in the production of particleboards without jeopardizing its performance. In SusB-bonded PBs, the adhesive amount can be reduced to 6.3% at a press factor of 10 s/mm, as sufficient IB values of 0.44 ± 0.10 N/mm^2^ are still reached with these production parameters. The IB is reduced by half (0.22 ± 0.04 N/mm^2^) when the resin content is further reduced to 4.5%. The mean IB values show a linear correlation with the SusB adhesive amount (R^2^ = 0.898) in the investigated range, having a Pearson correlation coefficient of 0.95.

**Figure 5:**
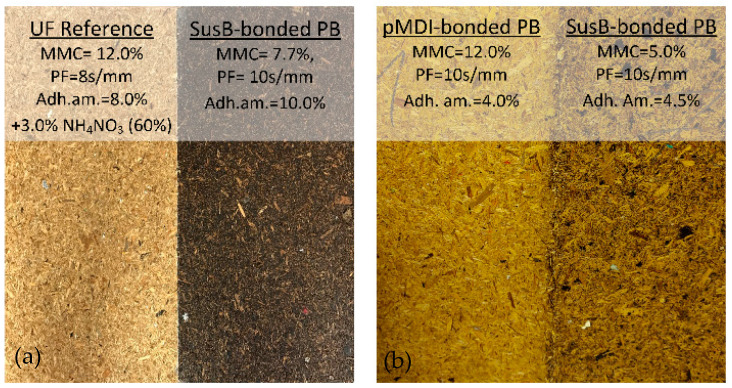
(**a**) Comparison of SusB-bonded particleboards with high adhesive amount to UF reference, (**b**) comparison of SusB-bonded particleboards with low adhesive amount to PMDI reference.

So far, the color of the SusB-bonded particleboards was not in the focus of the development and optimization. However, a qualitative examination of the produced boards showed that SusB-bonded PBs with reduced adhesive amount are clearly brighter in color, although not as light as pMDI-bonded particleboards (Figure 5). Further optimization of the color might be a desirable aspect for future work. 

#### 3.3.4. Thickness Swelling and Water Absorption

**Figure 6:**
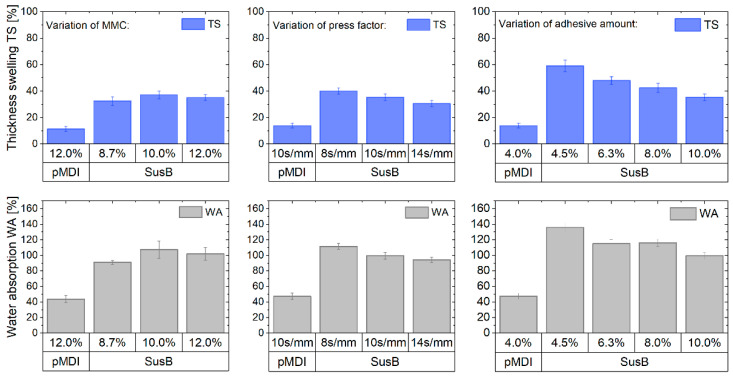
Dimensional stability of the single-layered particleboards: Thickness swelling after 24 h of immersion in water and springback (**upper** plots) as well as water absorption (**lower** plots), *n* = 20.

The 24 h thickness swelling (TS) and water absorption (WA) of the single-layered particleboards are depicted in Figure 6. As can be seen in the upper middle plot, the maximum thickness swelling of SusB-bonded PBs is in the range of 30.6 ± 2.4% (press factor = 14 s/mm) and 40.1 ± 2.4% (press factor = 8 s/mm). Higher press factors give the adhesive more time to form a sufficient network and develop cohesive strength. This result was already indicated in the preliminary adhesive testing (Figure 2, right plot) and underlined by the internal bond strength evaluation (Figure 4, middle plot). A surprising finding is that the 24 h TS of these SusB-bonded boards is actually quite comparable to UF-bonded boards. Papadopoulos [40] found a 24 h TS of 20–30% for particleboards bonded with a 7.5–10% resin amount (board thickness 12.15 mm, density 650 kg/m^3^, press factor 14 s/mm). In general, TS and WA improve when the adhesive amount increases. This trend was also reported for urea-formaldehyde- and phenol-formaldehyde-bonded particleboards [40,45], and as can be seen in Figure 6 (upper right plot), it is also applicable for SusB-bonded boards. Typically, it is a result of improved interparticle bonding, which positively affects the thickness stability. 

An interesting finding is that no significant differences in TS between particleboards with MMC = 8.7–12% can be seen in Figure 6 (left plot). Nevertheless, the water absorption results (Figure 6, lower plots) show quite clearly that the present SusB adhesive is not suitable for use in humid conditions. The maximum thickness swelling (24 h) for non-load-bearing boards in humid conditions (EN 312, P3 classification [31]) is 14%. The obtained TS of the SusB boards is almost twice as high as for the pMDI-bonded ones. The SusB adhesive has a high renewable content of 80 mol.-% (without solvent), with fructose being the main compound. As such, it is very likely that many polar functional groups remain available in the resin, leading to a high affinity for water uptake. This is a clear disadvantage, which is well-known for other carbohydrate-based adhesives. Carbohydrates such as fructose, glucose, sucrose or xylose contain large numbers of hydroxyl groups, which cause water sensitivity. This is also a reason for why carbohydrates are not typically used in wood bonding [27]. The SusB adhesive was developed for boards used for interior fitments used in dry conditions (EN312, P2 classification [31]) with the intention to completely substitute the typically used urea-formaldehyde adhesive, and no hydrophobizing agents have been used for the production. 

The results confirm that the SusB adhesive is a good choice for its intended application in dry conditions, but further research and development is necessary to overcome the limitation of dimensional stability after water immersion. Future topics for adhesive research should focus on the fundamental understanding of the bond failure criteria after water immersion and the interaction between wood and adhesive. Furthermore, it is well-known that the use of additives, e.g., wax emulsions or other hydrophobizing agents, improves the dimensional stability of UF-bonded particleboards. This is also an interesting future approach for SusB-bonded adhesives. Further dimensional stability testing is best carried out in three-layered particleboards. The wood particles used in the face layer are typically smaller [46], leading to a lower wood mass of each particle and an increased number of particle–particle interactions [12]. Previous publications hypothesize that this results in less internal swelling of the wood particles [12]. In addition, three-layered particleboards would be closer to the industrial application [46] and allow the development of different SusB adhesive compositions in the core and face layers.

## 4. Conclusions

The influence of several production parameters such as mat moisture content, adhesive amount and press factor on the physical and mechanical performance of single-layered particleboards made of sustainable fructose/HMF/BHMT (SusB) adhesive was investigated. On the basis of the conducted press series, it can be concluded that the mat moisture content has the highest impact on the IB of the produced board. It was found that the presence of higher amounts of water has a direct influence on the chemical curing reaction, as it promotes side reactions and reduces the reaction enthalpy of the main curing peak from 371.9 J/mol to 270.5 J/mol. 

Higher press factors (10 s/mm, 14 s/mm) improve the IB as well as the dimensional changes of the board after 24 h of immersion in water. It is important to point out that SusB-bonded PBs made at 10 s/mm (MMC = 7.7%) produce similar results in terms of IB as pMDI-bonded ones with 0.59 ± 0.12 N/mm^2^ and 0.59 ± 0.09 N/mm^2^. The mean thickness swelling of the aforementioned boards is, however, twice as high for the SusB-made boards, with 30.6 ± 2.4%. In future work, investigating the effect of additives such as wax emulsions on the dimensional stability after water immersion might prove important.

In terms of internal bond strength, both pMDI- and SusB-based particleboards are comparable to conventional UF-based systems. For a summarizing conclusion, a life cycle assessment and techno-economic analysis of the herein studied adhesives are planned and certainly required for giving direction to future developments of formaldehyde-free alternatives. 

## Figures and Tables

**Table 1:** Press series 1—Particleboards with varied board moisture content. Dry wood = 100%.

Adhesive	pMDI Reference	SusB	SusB	SusB
Amount of adhesive (on dry base) [%]	4.5	10	10	10
Moisture content of wood particles [%]	3.0	3.0	3.0	3.0
Added water based on dry wood [wt.%]	11.2	4.6	1.8	0.0
Resulting initial mat moisture content [wt.%]	12.0	12.0	10.0	8.7
Press factor [s/mm]	8	14	14	14

**Table 2:** Press series 2—Particleboards with varied press factor and adhesive amount.

Adhesive	pMDI Reference	SusB	SusB	SusB	SusB	SusB	SusB
Amount of adhesive [%]	4	10	10	10	4.5	6.3	8.0
Moisture content of wood particles [%]	1.8	1.8	1.8	1.8	1.8	1.8	1.8
Mat moisture content (MMC) [wt.%]	12.0	7.7	7.7	7.7	5.0	5.7	6.6
Added water based on dry wood [wt.%]	12.4	0.0	0.0	0.0	0.0	0.0	0.0
Press factor [s/mm]	10	14	10	8	10	10	10

**Table 3:** Characteristic temperatures and reaction enthalpy of deconvoluted DSC peaks of SusB adhesive with and without additional water.

**SusB Adhesive**			
**Peak [-]**	**Onset Temperature [°C]**	**Peak Temperature [°C]**	**Enthalpy [J/g]**
1	70.7	84.3	2.66
2	82.7	117.7	371.88
3	118.3	123.6	2.66
4	145.0	156.5	5.46
5	149.5	174.1	82.34
**SusB Adhesive + Added Water**			
**Peak [-]**	**Onset Temperature [°C]**	**Peak Temperature [°C]**	**Enthalpy [J/g]**
1	73.0	91.3	33.74
2	74.7	113.7	270.46
3	113.0	123.6	6.61
4	149.2	166.9	8.70
5	82.5	87.1	0.38

## Supplementary Materials

The following supporting information can be downloaded at: https://www.mdpi.com/article/10.3390/ma15238701/s1, Table S1: Pearson coefficient: Density-IB relation for unconditioned and conditioned particleboards of press series 1 (mat moisture content variation); Figure S1: Density of the produced particleboards from press series 1 & 2, Target density = 650 kg/m^3^; Table S2: Turkey’s Post hoc test: Particleboards with a significant difference in density at a confidence interval of 95%. Table S3: Wood particle size distribution.
