# Supplementary material for: Effect of Mat Moisture Content, Adhesive Amount and Press Time on the Performance of Particleboards Bonded with Fructose-Based Adhesives
Source: Materials (Basel). 2022 Dec 6;15(23):8701. doi: 10.3390/ma15238701 (PMC9735780; doi:10.3390/ma15238701)
Supplement: Supplementary file 1 [file materials-15-08701-s001.zip › materials-2040822-supplementary.pdf]

Supplementary Materials

Effect of Mat Moisture Content, Adhesive Amount and Press Time on the Performance of Particleboards Bonded with Fructose-Based Adhesives

1. Pearson Correlation coefficient

Table S1. Pearson coefficient: Density- IB relation, for unconditioned and conditioned PB of Press series 1 (Mat moisture content variation).

Particleboard (Press Series 1)	Pearson Coefficient (95% Confidential Interval)
SusB_12%_cond._1	0.90358
SusB_12%_cond._2	0.37361
SusB_10%_cond._1	0.53561
SusB_10%_cond._2	0.36308
SusB_8.7%_cond._1	0.72924
SusB-8.7%_cond._2	0.39686
**Particleboard (Press Series 2)**	**Pearson Coefficient (95% Confidential Interval)**
PMDI_10s/mm_cond _1	0.84563
PMDI_10s/mm_cond _2	0.95459
SusB_10s/mm_cond._1	0.91429
SusB_10s/mm_cond._2	0.91378
SusB_14s/mm_cond._1	0.9170
SusB_14s/mm_cond._2	0.8265
SusB_8s/mm_cond._1	0.86243
SusB_8s/mm_cond._2	0.65301

2. Turkey's Post Hoc Test

Density of produced particleboards (each boxplot contains the data from two boards):

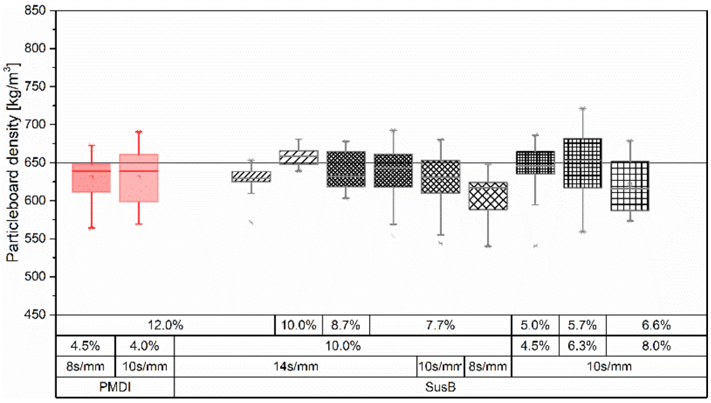

Figure S1. Density of the produced particleboards from press series 1 & 2, Target density=650 kg/m³.

Table S2. Turkey's Post hoc test: Particleboards with a significant difference in density at a confidence interval of 95%.

Adhesive/Press Factor/ Adh.Amount/ MMC	Mean Difference	Standard Error	q-Value	*p*-Value	Significance	95% Confidence Interval	
						Lower Bound	Upper Bound
SusB/14s/mm/10%/10% SusB/10s/mm/8.0%/6.6%	37.39	10.62	4.977	0.022	significant	2.81	71.96
SusB/14s/mm/10%/10% SusB/8s/mm/10%/7.7%	52.10	10.62	6.93548	9.8E-5	Significant	17.52	86.67
SusB/8s/mm/10%/7.7% SusB/10s/mm/6.3%/5.7%	39.66	10.62	5.279	0.011	significant	5.08	74.23

Table 3. Wood particle size distribution.

Sieve size mm	Sieve size mm	Sieve size mm	Sieve size mm	Sieve size mm	Sieve size mm	Sieve size mm	Sieve size mm	Sieve size mm
8.0	4.0	2.0	1.4	1.0	0.8	0.4	0.2	<0.2
Particle mass g	Particle mass g	Particle mass g	Particle mass g	Particle mass g	Particle mass g	Particle mass g	Particle mass g	Particle mass g
1.79	41.24	132.15	27.38	41.47	12.19	31.89	11.03	5.07
